# Down-regulation of FTX promotes the differentiation of osteoclasts in osteoporosis through the Notch1 signaling pathway by targeting miR-137

**DOI:** 10.1186/s12891-020-03458-0

**Published:** 2020-07-13

**Authors:** Yingfeng Yu, Peiquan Yao, Zhikun Wang, Wenwei Xie

**Affiliations:** Department of Orthopedics, the Third People’s Hospital of Dongguan City, No.1 Xianglong Road, Huangzhou, Shilong Town, Dongguan, 523326 Guangdong Province China

**Keywords:** FTX, Osteoclasts, Notch1 signaling pathway, miR-137

## Abstract

**Background:**

Osteoporosis (OP) is one of the commonly seen bone diseases with low bone mineral densities and trauma fractures. Accumulative studies have demonstrated that the occurrence of OP is closely related to osteoclasts differentiation. LncRNA FTX has been demonstrated to inhibit the development of some human cancers. However, its potential functions in human OP remains to be elusive.

**Methods:**

The expressions of FTX and miR-137 in bone and serum samples of patients with or without OP were measured. Bioinformatics analysis, RIP assays and luciferase reporter assays were performed to examine the upstream and downstream transactional factors of miR-137. Functional assays were conducted to check the roles of the Notching1 signaling pathway OP.

**Results:**

FTX was suppressed in OP samples and serums, however, miR-137 was greatly elevated. FTX reduced osteoclast-genesis and inhibited osteogenic differentiation by targeting miR-137. This also inhibited the Notch1 signaling pathway.

**Conclusion:**

Our experiments and results pointed out that lncRNA FTX up-regulated miR-137 in OP through the Notch1 signaling pathway.

## Background

Osteoporosis (OP) is one of the commonly seen bone diseases with low bone mineral densities and trauma fractures [12, 13]. A large population suffers from OP, therefore, it possesses a great burden on both public health and the economy [18]. Accumulative studies have demonstrated that the occurrence of OP is closely related to osteoclasts differentiation [26]. In recent years, long non-coding RNAs (lncRNAs) and microRNAs (miRNAs) are attracting more and more interest. These two non-coding RNAs may contribute to the dysregulated osteoclasts differentiation [8]. Therefore, biologists are paying more effort in the study of lncRNAs and miRNAs in the diagnostics, treatment directions, and prognostics for patients with OP.

LncRNAs are a group of RNA molecules with lengths of over 200 nucleotides, while they cannot be translated to proteins [11]. There is growing evidence suggesting that lncRNAs act as essential roles in a series of biological procedures including transcriptional regulations, cell apoptosis, as well as tumorigenesis [2, 6]. For OP, lncRNAs were proved to participate in the differentiation of osteoclasts. Long non-coding RNA FTX is a conserved functional lncRNA that could encode in the X-inactivation center [28]. LncRNA FTX has been demonstrated to inhibit the development of hepatocellular carcinoma [28], cardiomyocyte [17], and colorectal cancer [7]. However, its potential functions in human OP remains to be elusive.

miR-137 is a critical member of the miRNAs family, which has a length of around 20 nucleotides [20, 22]. Early in the year of 2008, J. Silber et al. indicated that miR-137 inhibited the proliferation of glioblastoma multiforme cells and induced differentiation of brain tumor stem cells [20]. There is also other literature revealing that miR-137 is playing an important role in a variety of human cancers [16, 29]. In our preliminary experiments based on bioinformatics, we found that lncRNA FTX might bind with miR-137 in OP. Therefore, we are inspired to investigate whether FTX and miR-137 play a combined role in the development of OP. Previous studies have indicated that Notch signaling pathways have crucial roles in osteoclasts differentiation [14, 15]. Herein, we also examined if this signaling is involved in the development of OP. Thorough experiments and analysis were carried out to investigate the associations among lncRNA FTX, miR-137, and Notch1 signaling pathway.

## Methods

### Primary human samples

Patients with hip fractures at the third People’s Hospital of Dongguan City were enrolled in this study, who were divided into 2 groups of OP patients and non-OP patients (control group). Osteoporosis were defined according to WHO criteria for classification of osteopenia and osteoporosis [12]. To be included, the fracture of the patients must meet the definition of fragility fracture caused by falling or low energy instead of violence or high-energy traumata. Patients with liver and kidney diseases; osteoarthritis; metabolic bone disease, ovarian diseases or other endocrine diseases were excluded. Also, patients underwent hormone therapy in recent 3 months or other chronic corticosteroid use were excluded due to the influence of these drugs on lncRNA and miRNA expression. Postmenopausal women with OP fractures (*n* = 40) and healthy volunteers (*n* = 40) were selected, who received hip replacement surgery in the hospital. Supplementary file 2 showed the patients’ background regarding their age, gender, and BMI. RNA was extracted from the whole bone. According to the T-score of BMD, the patients were divided into two groups: the normal group (T-score ≥ − 1.0) and the OS group (T-score ≤ − 2.5). Serum and bone samples were placed in endoprosthesis. All patients signed informed consent. This research was approved by the Ethics Committee of the third People’s Hospital of Dongguan City.

### CD14 + PBMC culture

Peripheral blood mononuclear cells (PBMC) had extraction according to Sorensen et al. [21]. CD14 antibody magnetic cell sorting (Miltenyi, Germany) was utilized to purify CD14 + PBMCs. CD14 + PBMCs were seeded in at 250, 000 cells/well. Cells were growing in alpha minimum essential medium (Invitrogen, USA) with 10% FBS (Gibco, CA), 50 IU/mL penicillin, and 50 mg/mL streptomycin, at 37 °C with humidity and 5% CO_2_. The culture medium was added in 25 ng/mL MCSF and 25 ng/mL RANKL (R&D Systems, USA).

### Cell transfections

FTX was amplified and purified. Then, pcDNA3.1 and FTX established the pcDNA-FTX recombinant plasmid and cloned to *E. coli*. The positive clones were selected and amplified. Next, recombinant plasmids were extracted and transfected to CD14 + PBMCs. For over-expression miR-137, CD14 + PBMCs were transfected with miR-137 mimic, or controls with Lipofectamine 2000 (Invitrogen, USA). All plasmids were made from Genepharma, China. At 48 h post-transfection, the cells were used for following experiments.

### Cell proliferation assays

4, 000 cells were seeded to a well from 96-well plate and cultured in alpha minimum essential medium with 10% FBS and antibiotics. 10 μL CCK-8 was added. We observed the signals at 2 h after the culture at 450 nm.

### qRT-PCR

Total RNA was extracted by TRIzol (Invitrogen, USA). 0.5 μg RNA was reverse-transcribed by PrimeScript (Takara Bio, Japan). 1 μg cDNA was employed to detect mRNA expressions by qRT-PCR with SYBR Kit (Takara Bio, Japan). GAPDH and U6 were regarded as references. Primers sequences are in the below:

GAPDH (forward: 5′-TGGATTTGGACGCATTGGTC-3′ and reverse: 5′-TTTG.

CACTGGTACGTGTTGAT-3′);

TNFRSF1B (forward: 5′-CGGGCCAACATGCAAAA.

GTC-3′ and reverse: 5′-CAGATGCGGTTCTGTTCCC-3′);

ACP5 (forward: 5′-GACT.

GTGCAGATCCTGGGTG-3′ and reverse: 5′-GGTCAGAGAATACGTCCTCAAA.

G-3′);

MMP-9 (forward: 5′-TGTACCGCTATGGTTACACTCG-3′ and reverse: 5′-GG.

CAGGGACAGTTGCTTCT-3′);

MMP-2 (forward: 5′-TGACTTTCTTGGATCGGGT.

CG-3′ and reverse: 5′-AAGCACCACATCAGATGACTG-3′);

TRAP (forward: 5′-TC.

ACCCTGACCTATGGTGC-3′ and reverse: 5′-GCCGGACTCCAATGTTAAAGC-3′);

cathepsin K (forward: 5′-CTGGCTGGGGTTATGTCTCAA-3′ and reverse: 5′-GGCT.

ACGTCCTTACACACGAG-3′).

### Luciferase reporter assays

FTX-WT (wild-type), FTX-MUT (mutant), Notch1-WT, Notch1-MUT were designed via the clone of WT 3′UTR or MUT 3′UTR to pMir-reporter vector (Ambion, USA). CD14 + PBMCs had co-transfections with luciferase plasmid, miR-137 mimic or miR-NC by Lipofectamine 2000. Dual-luciferase reporter assays (Promega, USA) were used to identify their binding effects.

### RNA pull-down assays

For pull-down assay, miR-137 without complementary sites with Notch1 was seen as internal reference (termed NC). MiR-137, miR-137-Mut, and NC were labeled with biotin to generate Bio-miR-137, BiomiR-137-Mut, and Bio-NC by GenePharma Company (Shanghai, China). Thereafter, they were transfected into CD14 + PBMCs. Forty-eight hours later, cells were harvested and incubated with Dynabeads M-280 Streptavidin (Invitrogen) for 10 min. After washing three times with buffer solution, the enrichments of bound RNAs were quantified and analyzed by qRT-PCR.

### RNA immunoprecipitation (RIP)

RIP assay was performed using the RNA-Binding Protein Immunoprecipitation Kit (Millipore). Firstly, we lysed CD14 + PBMCs and incubated them with AGO2 antibody or IgG. RNA-protein complexes were immunoprecipitated with protein A agarose beads. Next, we extracted the RNAs using Trizol (Invitrogen, USA). The IP-western was employed to measure AGO2 protein and qRT-PCR was conducted to detect the mRNA expression of FTX, using RIPAb+ Ago2-RIP Validated Antibody and Primer Set, 03–110, Merck Millipore, US.

### TRAP staining assay

Induced osteoclasts were fixed with 4% paraformaldehyde (PFA) for 20 min at room temperature and then stained by TRAP fluid for 1 h. TRAP solution was prepared by mixing acetate buer (pH 5.0), 50 mM sodium tartrate, naphthol AS-MX phosphate (Sigma Aldrich, Tokyo, Japan), and Fast Red Violet LB salt (Sigma Aldrich, Tokyo, Japan). After removal of the TRAP fluid, the plate was washed three times with phosphate-buffered saline (PBS). TRAP-positive multinuclear cells were observed using an inverted microscope.

### Resorption pit assay

Mature osteoclasts were generated by coculturing of mouse bone marrow cells and osteoblasts with VitD3 (10 nM) on collagen gels for 6 days. The generated osteoclasts were replated on an Osteo Assay Surface plate (Corning, NY, USA), allowed to settle for 2 h, and then incubated with different concentrations of WESS for 24 h. After the incubation period, osteoclasts were visualized by TRAP staining. Resorption pits were photographed and analyzed by using Image J software, after removing cells by using sodium hypochlorite bleach.

### Western blotting

Protein was separated by 10% SDS-PAGE (Thermo, USA) and sent to PVDF membrane (Millipore, USA). The membrane was blocked by 5% BSA. Next, we treated the membranes with anti-Notch1 (1:1000, Abcam, UK), anti-GAPDH (1:1000, beyotime, China) at 4 °C for a night and then HRP-anti-rabbit (1:1000, beyotime, China) for 2 h at 25 C. We detected the signals using the ECL (Bio-Rad Lab, USA).

### Flow cytometry

After differentiation, cells were collected, washed and suspended. Then, it was incubated by Annexin V at 37 °C for 10 min, and stained by propidium iodide. Flow cytometer was employed to measure cell apoptosis.

### Statistical analysis

All results were shown as mean ± SD, analyzed by SPSS 21.0. Differences between groups were conducted by Student’s t-test (2 groups) and one-way ANOVA (> 2 groups). *P*-value of < 0.05 was considered to have significance.

## Results

### FTX was down-regulated and miR-137 was up-regulated in osteoporotic samples

**Fig. 1:**
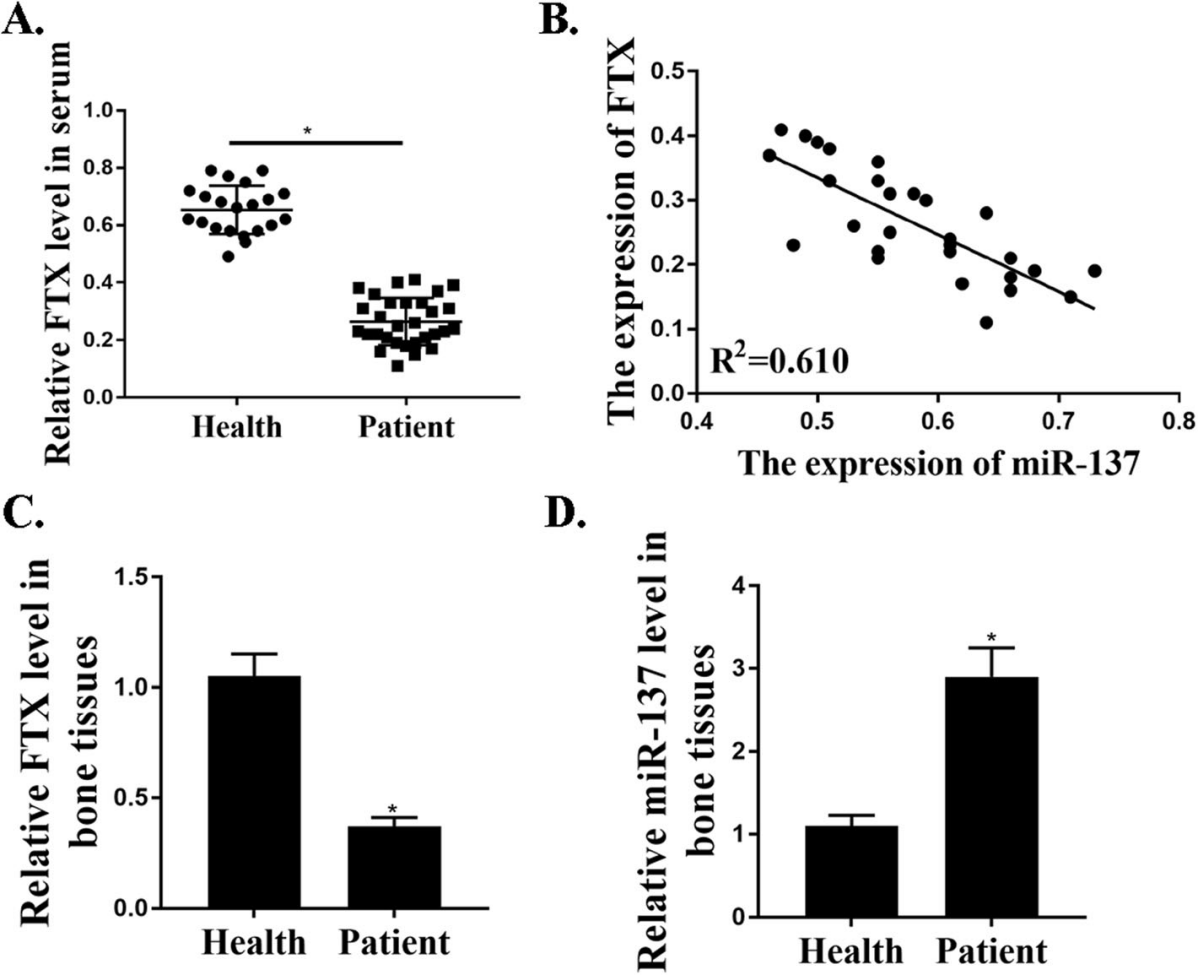
FTX was down-regulated and miR-137 was up-regulated in osteoporotic samples. **a**. The FTX expressions in the serum of non-OP (healthy) (*n* = 21) and OP patients (*n* = 30). **b**. The relationship between FTX and miR-137 expressions in the serum of OP patients. **c**. The FTX expressions in the bone tissues of non-OP (healthy) (*n* = 7) and OP patients (*n* = 7). D. The miR-137 expressions in the bone tissues of non-OP (healthy) (*n* = 7) and OP patients (*n* = 7). *p < 0.001

The expressions of FTX in serum and bone tissues of OP patients were analyzed. The expressions of FTX in patients’ serum (*n* = 30) were down-regulated compared with control (*n* = 21) (Fig. 1a). It is negatively related to the miR-137 expressions (Fig. 1b). In addition, the expressions of FTX in patients’ bone tissues (*n* = 7) were down-regulated compared with control (non-OP) (Fig. 1c) (*n* = 7). miR-137 was up-regulated in OP patients’ bone tissues (Fig. 1d).

### Over-expressions of FTX inhibited osteoclast differentiation

**Fig. 2:**
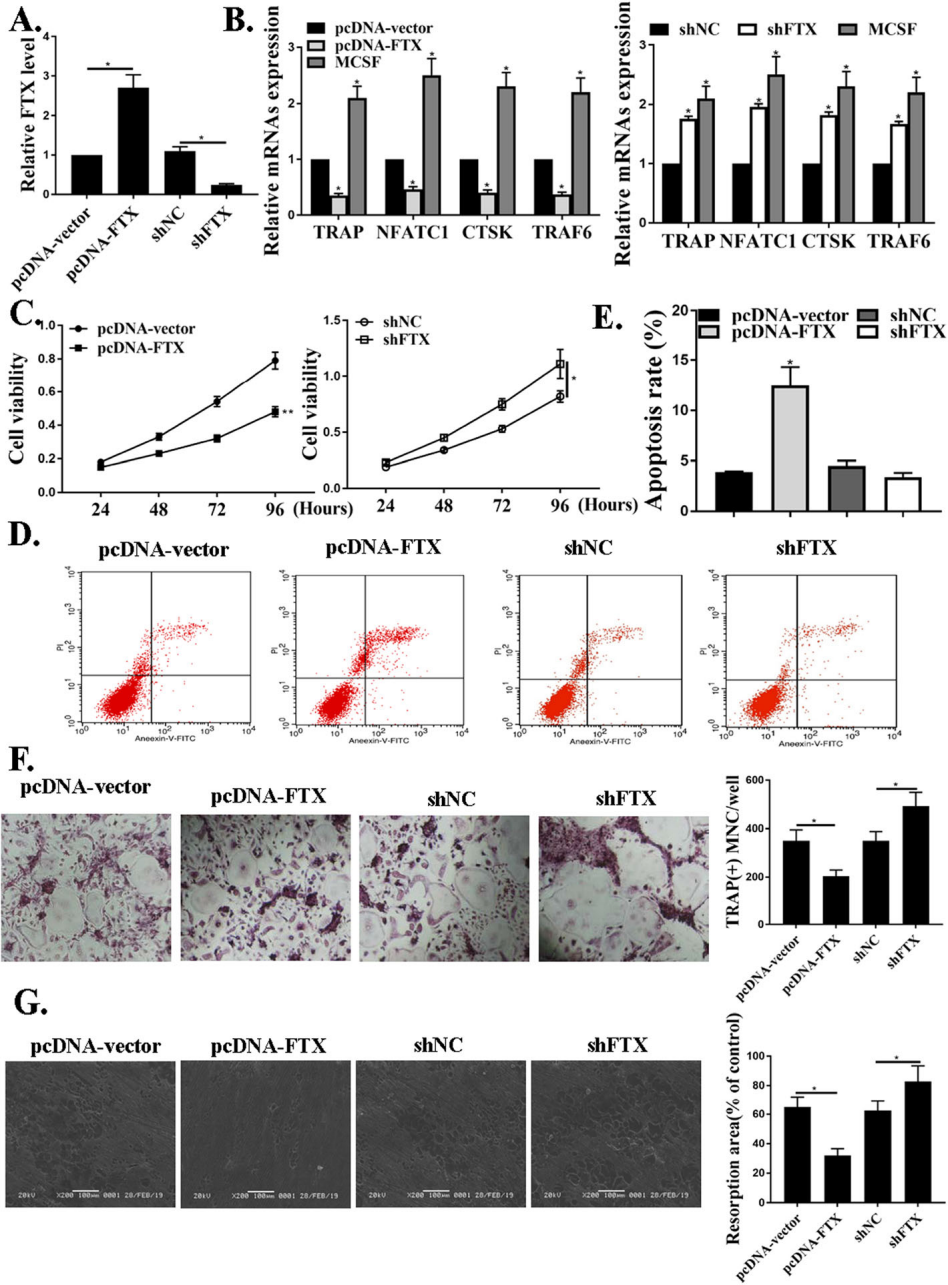
Over-expressions of FTX inhibited osteoclast differentiation. **a**. FTX mRNA levels under pcDNA-vector or pcDNA-FTX. **b**. qRT-PCR results of the expressions of TRAP, CTSK, and NFATC1, TRAF6 under pcDNA-vector or pcDNA-FTX. **c**, **d**, and **e**. Cell viability and cell apoptosis of CD14 + PBMCs in transfection with pcDNA-vector or pcDNA-FTX. **f**. TRAP-staining photos for osteoclast differentiation under transfection with pcDNA-vector or pcDNA-FTX. *P < 0.001. *n* = 3

pcDNA-FTX was efficiently over-expressed successfully in CD14 + PBMCs, according to Fig. 2a. Figure 2b showed that osteoclast marker of TRAP, NFATC1, CTSK, and TRAF6 were hindered by FTX. Figures 2c-e demonstrated that the viabilities of CD14 + PBMCs were decreased by FTX, but cell apoptosis rate was promoted under FTX over-expressions. Figure 2f showed the experiments from TRAP-stained osteoclasts from each group, which revealed that pcDNA-FTX greatly inhibited osteoclast differentiation. It was obvious that FTX regulated osteoclast differentiation in CD14 + PBMCs.

### miR-137 was one of the targets of FTX in CD14 + PBMCs

**Fig. 3:**
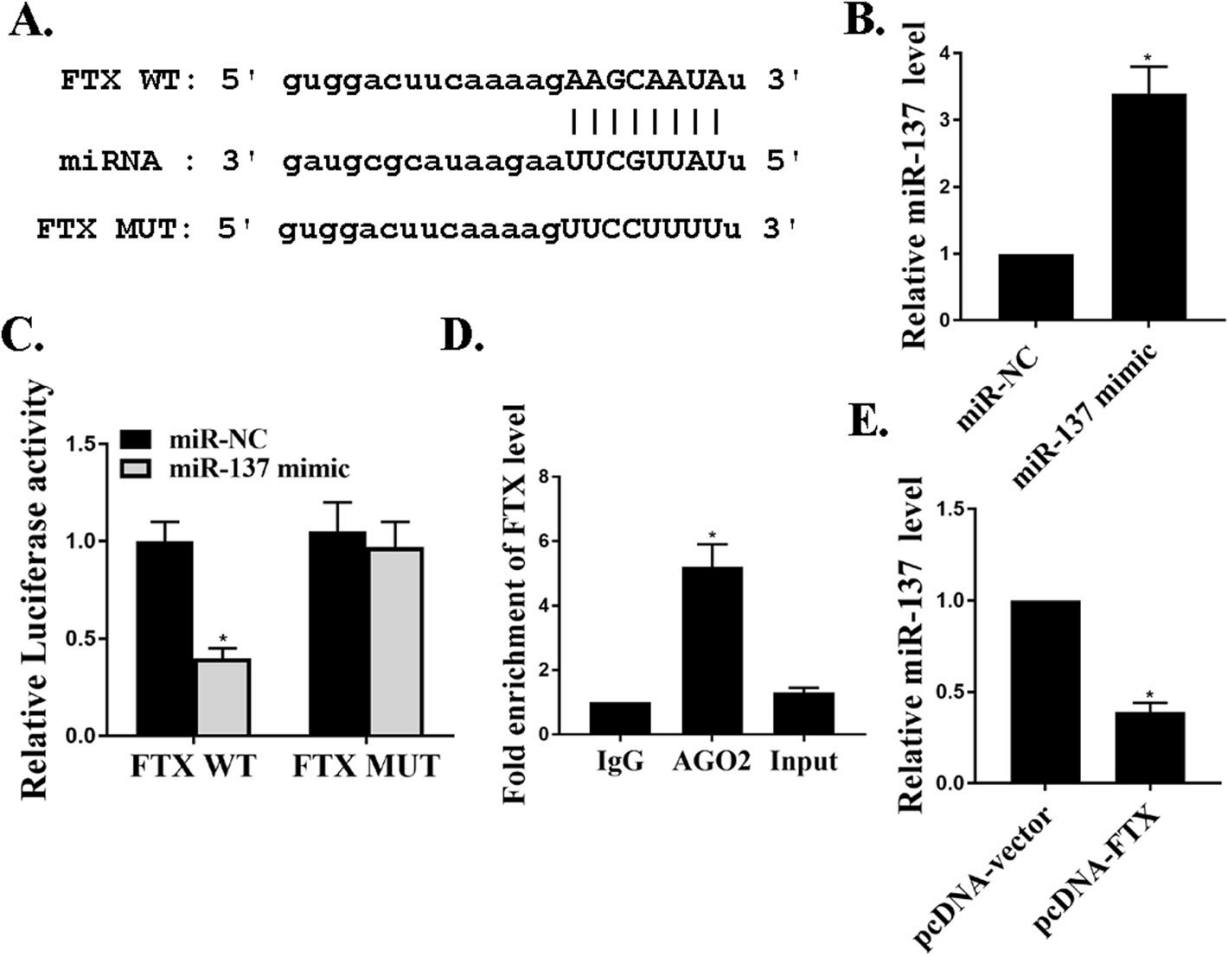
miR-137 was one of the targets of FTX in CD14 + PBMCs. **a**. The binding sites between miR-137 and FTX by Starbase. **b**. The miR-137 mRNA. **c**–**d**. Luciferase reporter assays and RIP assays for the interactions between miR-137 and FTX. **e**. The miR-137 mRNA expressions under the transfection of FTX. *p < 0.001, *n* = 3

Figure 3a predicted the shared binding sites between FTX and miR-137 using bioinformatics. It also showed the relevant sequences of FTX-WT, miR-137, and FTX-MUT. Figure 3b investigated the successful transfection of miR-137 in cells. Next, we conducted the experiments to study the interactions between miR-137 and FTX in OP by luciferase reporter assays and RIP assays in CD14 + PBMCs. RIP is a powerful method to investigate the physical associations between proteins and RNA molecules. Here, we utilized RIP experiment to verify the binding effect between FTX and miRNA. From Fig. 3c, miR-137 over-expressions resulted in a lower activity of FTX-WT. Figure 3d showed the RIP for binding effects between FTX and miR-137 with the AGO2 antibody. In contrast to the control IgG, higher FTX levels were found in the AGO2 antibody. Figure 3e revealed that over-expressions of FTX could down-regulate miR-137 mRNA expressions in CD14 + PBMCs.

### miR-137 mediated the effects of FTX on osteoclast differentiation

**Fig. 4:**
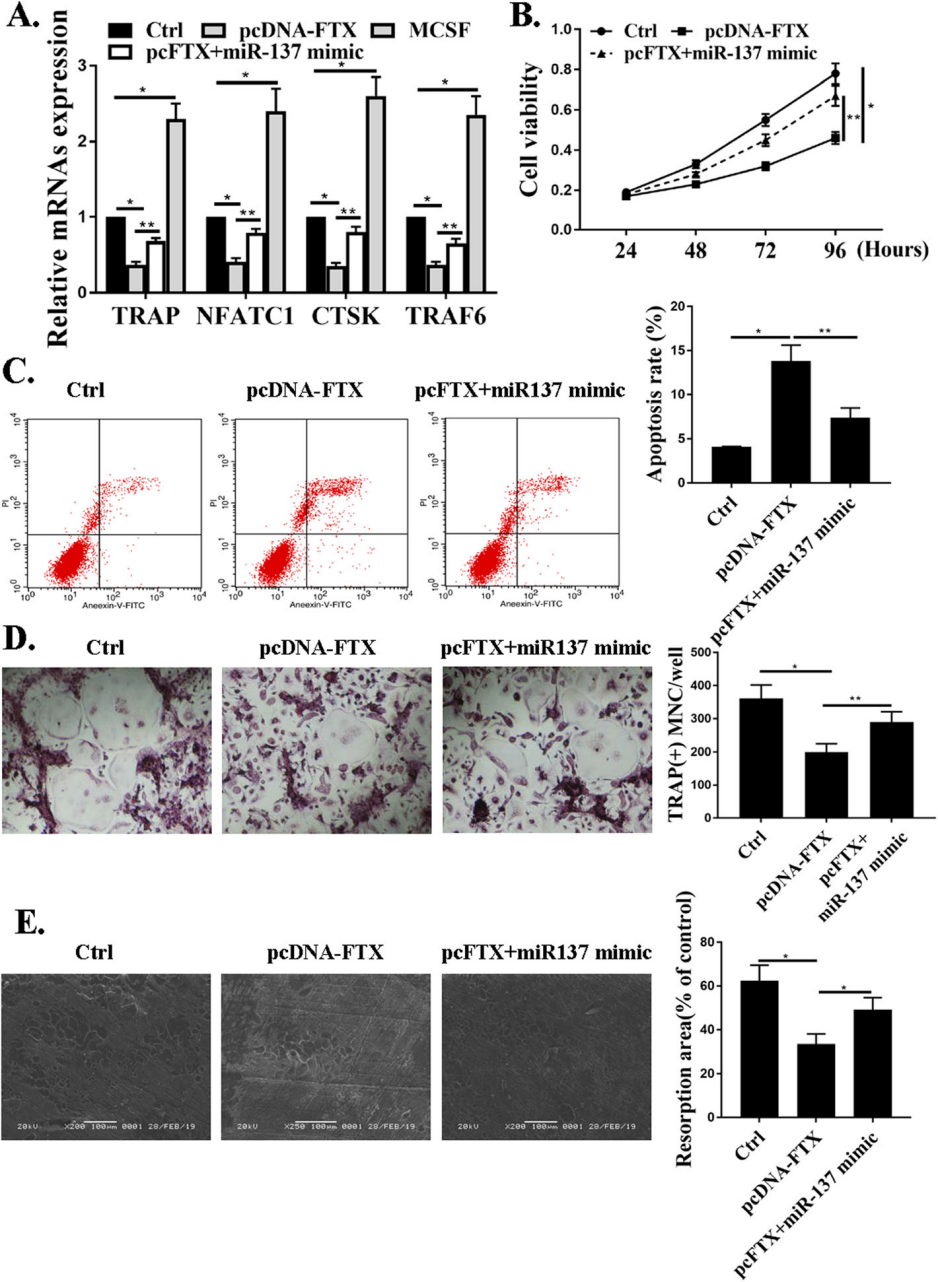
miR-137 mediated the effects of FTX on osteoclast differentiation. **a**. qRT-PCR for expressions of TRAP, CTSK, and NFATC1, TRAF6 CD14 + PBMCs. **b** and **c**. Cell viability and apoptosis by CCK-8 assays and flow cytometry. **d**. TRAP-staining photos for osteoclast differentiation under transfection with pcDNA-FTX or pcFTX+miR-137 mimic. **e**. Representative microscopic pictures of resorption pit*P < 0.001. **P < 0.001, *n* = 3

According to Fig. 4a, osteoclast-specific genes of TRAP, NFATC1, CTSK, and TRAF6 were all inhibited by FTX suppression, but this result was blunted by a miR-137 mimic. Figure 4b and c revealed that the viabilities of CD14 + PBMCs were mitigated by FTX but the effects were blunted by a miR-137 mimic. Cell apoptosis was promoted by FTX but the effects were blunted by a miR-137 mimic. Supplementary figure 1A showed the apoptotic rate for early apoptotic cells (LR), and late apoptotic cells (UR). It was noticed that late apoptotic cells appeared under the transfection of pcDNA-FTX. However, this effect was also attenuated by miR-137 (supplementary figure 1B). Figure 4d showed the experiments from TRAP-stained osteoclasts from each group, which revealed that pcDNA-FTX greatly inhibited osteoclast differentiation, while miR-137 mimic attenuated this effect. The data pointed out that FTX regulated osteoclast differentiation in CD14 + PBMCs.

### MiR-137 regulated the Notch1 signaling pathways in CD14 + PBMCs

**Fig. 5:**
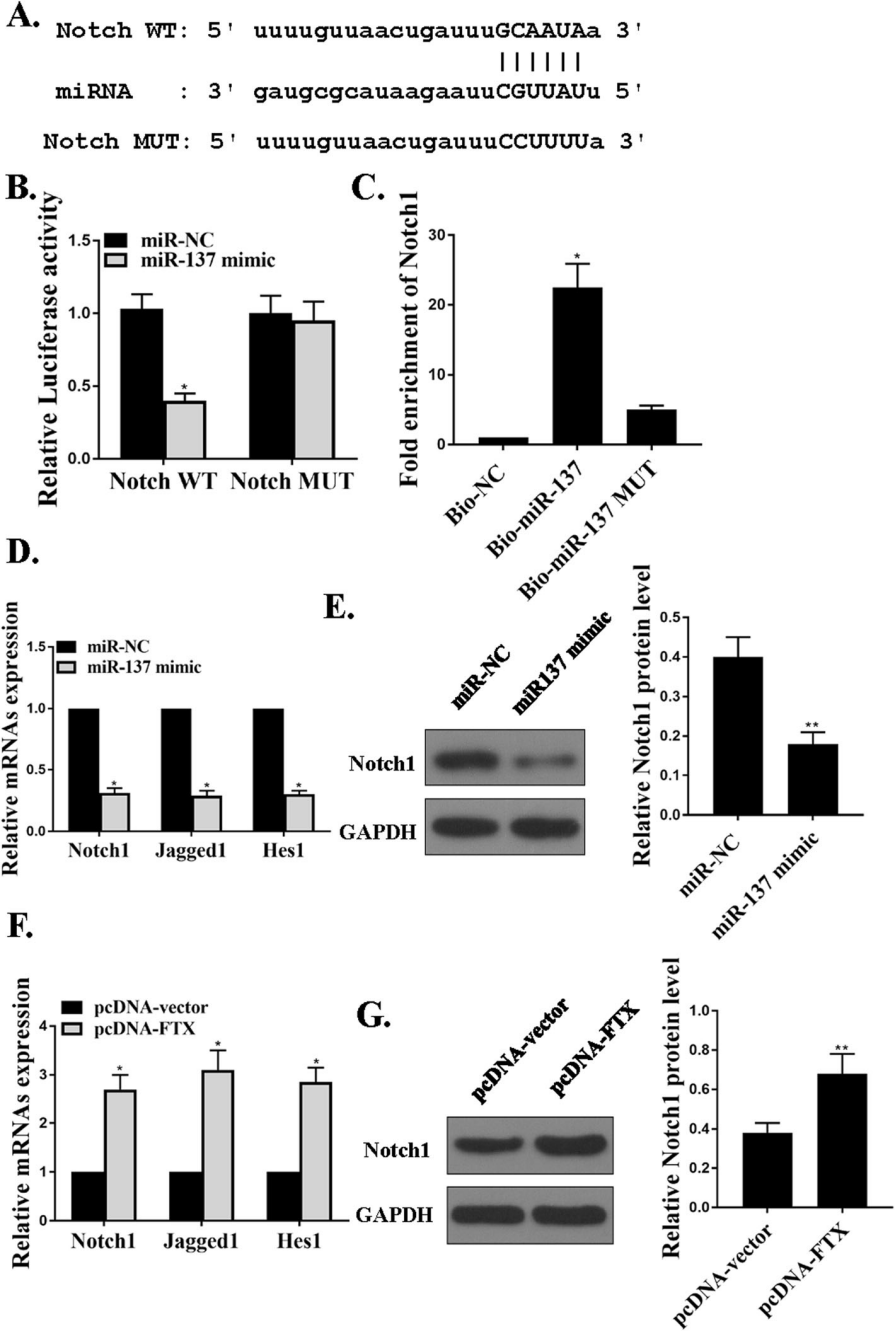
MiR-137 regulated the Notch1 signaling pathways in CD14 + PBMCs. **a**. The binding sites in miR-137 and Noch1. **b** and **c**. Luciferase assays and RNA pull-down assays for interactions between miR-137 and Notch1. **d** and **e**. The Notch1 mRNA and protein expressions under miR-137 mimic. **f** and **g**. Notch1 mRNA and protein expressions transfection of FTX. *p < 0.001, **P < 0.05, *n* = 3

Figure 5a predicted the binding site in Notch1 and miR-137 using bioinformatics. It showed the relevant binding sequences of Notch-WT, miR-137, and Notch-MUT. From Fig. 5b, miR-137 over-expressions resulted in the decreased luciferase activity of Notch1-WT. Figure 5c showed the pull-down assays. We found that Notch1 was pulled-down via bio-miR-137 but not bio-NC or bio-miR-137-Mut. Figure 5d and e demonstrated that over-expressions of miR-137 could down-regulate Notch1 mRNA and protein expressions in CD14 + PBMCs, but over-expressions of FTX could up-regulate Notch1 these expressions.

### The effects of miR-137 on osteoclast differentiation were mediated by Notch1 signaling pathways in CD14 + PBMCs

**Fig. 6:**
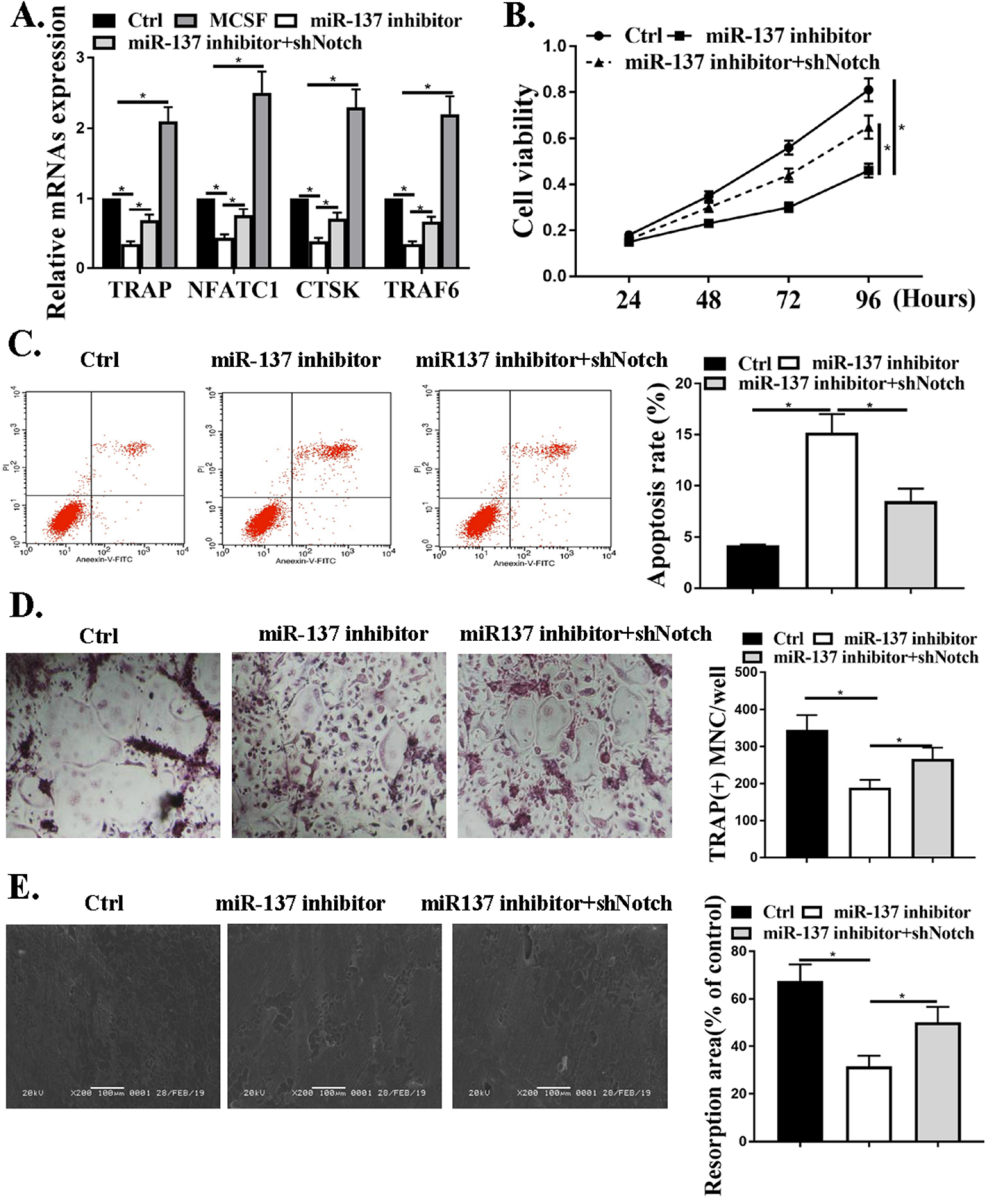
The effects of miR-137 on osteoclast differentiation were mediated by Notch1 signaling pathways in CD14 + PBMCs. **a**. qRT-PCR for expressions of TRAP, CTSK, and NFATC1, TRAF6 CD14 + PBMCs. **b** and **c**. Cell viability and apoptosis by CCK-8 assays and flow cytometry. **d**. TRAP-staining photos for osteoclast differentiation under transfection with miR-137 inhibitor or miR-137 inhibitor+shNotch. **e**. Representative microscopic pictures of resorption pits. *P < 0.001. **P < 0.001, *n* = 3

To further illustrate the mechanisms underlying miR-137 and osteoclast differentiation, we evaluated the relative genes and cells in this process. The osteoclast-specific genes of TRAP, NFATC1, CTSK, and TRAF6 were all inhibited by miR137 suppression, but this result was blunted by shNotch (Fig. 6a). Furthermore, the viabilities of CD14 + PBMCs were mitigated by mirR-137 but the effects were blunted by a miR-137 mimic (Figs. 6b & c). Cell apoptosis was promoted by FTX but the effects were blunted by shNotch. Figure 6d showed the experiments from TRAP-stained osteoclasts from each group, which revealed that mir137 inhibitor greatly inhibited osteoclast differentiation, while shNotch attenuated this effect. Resorption pit assay also showed the same results (Fig. 6e.) The data pointed out that miR-137-notch regulated osteoclast differentiation in CD14 + PBMCs.

## Discussion

Previous studies have established that OP probably leads to the dysregulation of some specific lncRNAs and miRNAs [3, 24, 25]. For example, Q. Wang et al. reported that the expressions of lncRNA MEG3 and miR-133a-3p were both up-regulated in OP samples [25]. Based on our qRT-PCR results, the expressions of FTX in patients’ serum was suppressed compared with control. Besides, it is negatively related to the expressions of miR-137. In addition, the expressions of FTX in patients’ bone tissues were suppressed compared with control (non-OP) and miR-137 was up-regulated in OP patients’ bone tissues. For the first time, we propose that FTX was suppressed and miR-137 was up-regulated in osteoporotic samples.

According to Q. Wang, the irregular expressions of related lncRNAs affect the osteogenic differentiation for OP patients [25]. Another study in 2018 also demonstrated that specific lncRNA could inhibit osteoclasts differentiation [23]. From our experiments, the osteoclast markers of TRAP, NFATC1, CTSK, and TRAF6 were all hindered by FTX. The viabilities of CD14 + PBMCs were decreased by FTX, but cell apoptosis rate was promoted under FTX over-expressions. It was obvious that FTX regulated osteoclast differentiation in CD14 + PBMCs. Similar to previous researches, we found that over-expressions of FTX inhibited osteoclast differentiation.

It was commonly known that lncRNAs and miRNAs could bind to each other and exert biological functions in multiple types of human cancers [5, 9]. Firstly, our study from bioinformatics predicted the shared binding sites between FTX and miR-137. The luciferase reporter assays and RIP assays also confirmed the close interactions between miR-137 and FTX in CD14 + PBMCs. Moreover, over-expressions of FTX could suppress miR-137 mRNA expressions in CD14 + PBMCs. As far as we know, we are the first to report that miR-137 was directly targeted by FTX in CD14 + PBMCs.

The interactions between lncRNAs and miRNAs may reverse the biological effects resulted from lncRNAs [10, 27]. In consistence with previous studies, we noticed that osteoclast-specific genes of TRAP, NFATC1, CTSK, and TRAF6 were all inhibited by FTX suppression, but this result was blunted by a miR-137 mimic. Moreover, the viabilities of CD14 + PBMCs were mitigated by FTX but the effects were blunted by a miR-137 mimic. Cell apoptosis was promoted by FTX but the effects were blunted by a miR-137 mimic. Consistent with the well-established theories, our studies revealed that FTX regulated osteoclast differentiation in CD14 + PBMCs, while miR-137 mediated the effects of FTX on osteoclast differentiation.

A variety of researches have demonstrated the essential effects of a Notch signaling pathway in both bone remodeling [1, 19] and OP [4, 14]. In our experiments, miR-137 over-expressions resulted in the decreased luciferase activity of Notch1-WT. Notch1 was pulled down by bio-miR-137 but not bio-NC or bio-miR-137-Mut. Over-expressions of miR-137 could suppress Notch1 mRNA and protein expressions in CD14 + PBMCs, but over-expressions of FTX could up-regulate Notch1 these expressions. As a novel finding, miR-137 regulated the Notch1 signaling pathway in CD14 + PBMCs.

## Conclusion

FTX was suppressed in OP samples and serums, however, miR-137 was greatly elevated. FTX reduced osteoclast-genesis and inhibited osteogenic differentiation by targeting miR-137, which inhibited the Notch1 signaling pathway. Our experiments and results pointed out that lncRNA FTX up-regulated miR-137 in OP by Notch1 signaling pathway.

## Supplementary information

**Additional file 1: Figure S1.** Apoptosis rate for UR and LR. A. The apoptotic rate for early apoptotic cells (LR), and late apoptotic cells (UR) under transfection of pcDNA-FTX and pcDNA-vector. B. The apoptotic rate for LR, and UR under transfection of pcDNA-FTX and pcFTX+miR-137 mimic.

**Additional file 2.**

**Additional file 3.**

## Data Availability

The analyzed data sets generated during the study are available from the corresponding author on reasonable request.

## Abbreviations

OP: Osteoporosis
lncRNAs: Long non-coding RNAs
miRNAs: microRNAs
