# Supplementary figures and images for: Down-regulation of FTX promotes the differentiation of osteoclasts in osteoporosis through the Notch1 signaling pathway by targeting miR-137
Source: BMC Musculoskelet Disord. 2020 Jul 13;21:456. doi: 10.1186/s12891-020-03458-0 (PMC7359489; doi:10.1186/s12891-020-03458-0)

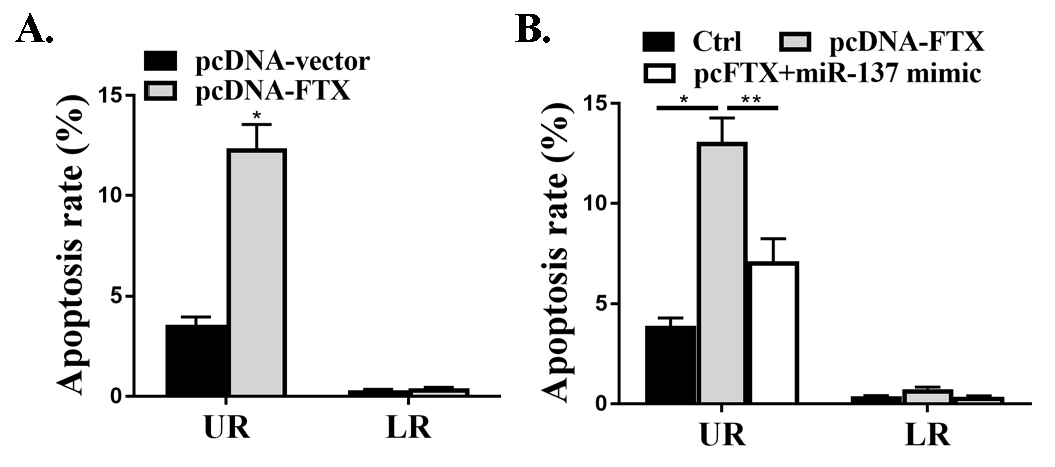

Supplement: Supplementary file 1 — Additional file 1: Figure S1. Apoptosis rate for UR and LR. A. The apoptotic rate for early apoptotic cells (LR), and late apoptotic cells (UR) under transfection of pcDNA-FTX and pcDNA-vector. B. The apoptotic rate for LR, and UR under transfection of pcDNA-FTX and pcFTX+miR-137 mimic. [file 12891_2020_3458_MOESM1_ESM.tiff]

Figure 5E Notch1、GAPDH


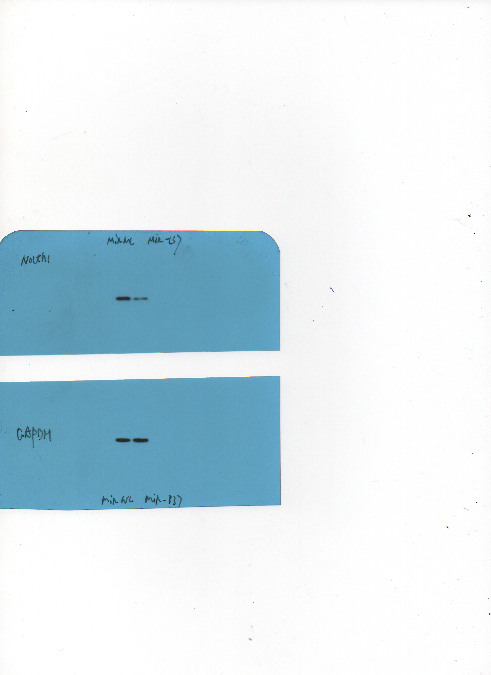


Figure 5G Notch1、GAPDH


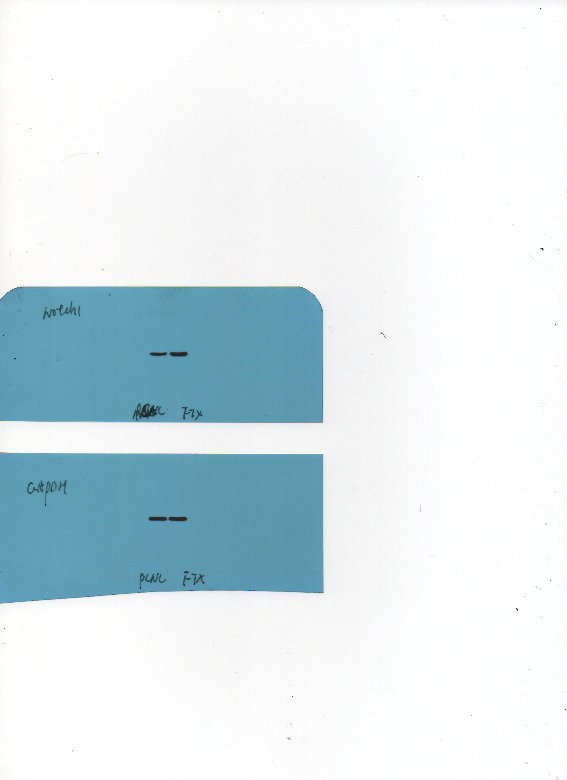

Supplement: Supplementary file 3 — Additional file 3. [file 12891_2020_3458_MOESM3_ESM.docx]
