# Supplementary material for: Down-regulation of FTX promotes the differentiation of osteoclasts in osteoporosis through the Notch1 signaling pathway by targeting miR-137
Source: BMC Musculoskelet Disord. 2020 Jul 13;21:456. doi: 10.1186/s12891-020-03458-0 (PMC7359489; doi:10.1186/s12891-020-03458-0)
Supplement: Supplementary file 2 — Additional file 2. [file 12891_2020_3458_MOESM2_ESM.docx]

**Parameter Health(Total21) Patient(Total30)**

**Age (years) 62+2.5 60.8+1.9**

Gender

Male 5 9

Female 16 21

BMI（kg/m2） 23.38**+1.8 24.72+2.1**
